# The case for investing in the male condom

**DOI:** 10.1371/journal.pone.0177108

**Published:** 2017-05-16

**Authors:** John Stover, James E. Rosen, Maria Nadia Carvalho, Eline L. Korenromp, Howard S. Friedman, Matthew Cogan, Bidia Deperthes

**Affiliations:** 1 Avenir Health, Glastonbury, Connecticut, United States of America; 2 United Nations Population Fund (UNFPA), New York City, New York, United States of America; RTI International, UNITED STATES

## Abstract

When used correctly and consistently, the male condom offers triple protection from unintended pregnancy and the transmission of sexually transmitted infections (STIs) and human immunodeficiency virus (HIV). However, with health funding levels stagnant or falling, it is important to understand the cost and health impact associated with prevention technologies. This study is one of the first to attempt to quantify the cost and combined health impact of condom use, as a means to prevent unwanted pregnancy and to prevent transmission of STIs including HIV. This paper describes the analysis to make the case for investment in the male condom, including the cost, impact and cost-effectiveness by three scenarios (low in which 2015 condom use levels are maintained; medium in which condom use trends are used to predict condom use from 2016–2030; and high in which condom use is scaled up, as part of a package of contraceptives, to meet all unmet need for family planning by 2030 and to 90% for HIV and STI prevention by 2016) for 81 countries from 2015–2030. An annual gap between current and desired use of 10.9 billion condoms was identified (4.6 billion for family planning and 6.3 billion for HIV and STIs). Under a high scenario that completely reduces that gap between current and desired use of 10.9 billion condoms, we found that by 2030 countries could avert 240 million DALYs. The additional cost in the 81 countries through 2030 under the medium scenario is $1.9 billion, and $27.5 billion under the high scenario. Through 2030, the cost-effectiveness ratios are $304 per DALY averted for the medium and $115 per DALY averted for the high scenario. Under the three scenarios described above, our analysis demonstrates the cost-effectiveness of the male condom in preventing unintended pregnancy and HIV and STI new infections. Policy makers should increase budgets for condom programming to increase the health return on investment of scarce resources.

## Introduction

The male and female condom are the only devices to offer protection against both unplanned pregnancy and the transmission of STIs including HIV. Couples have used the male condom (from this point, the term “condom” is used to refer to the male condom) for centuries. Analysts have made several attempts to separately estimate the effectiveness of condoms for preventing unplanned pregnancy and in preventing HIV and STIs as shown in Table 1. However, few studies have tried to measure the combined protection that condoms afford against all three conditions. UNAIDS recently set the goal of achieving 90% condom use during the most recent sexual activity with a non-regular partner. With international support for and investment in condom programming stagnating globally [1], it is critical to fully understand the importance of condoms as a global health intervention. Donors and national governments need evidence to help them strategically and cost-effectively determine how to invest in family planning (FP) and prevent STIs, including HIV. To meet this need, we conducted this analysis.

**Table 1:** Estimates of condom effectiveness.

Objective	Effectiveness at prevention	Source
Prevent unintended pregnancy	98%	Trussel J, 2011 [2]
Prevent HIV infection	80% proportionate reduction in HIV seroconversion with condom use	Weller et al., 2004 [3]
	Prevalence of HIV infection declined from 89% to 32% among female sex workers in Abidjan who used condoms	Ghys PD et al., 2002 [4]
Prevent STI infection	“Condom use is associated with statistically significant protection of men and women against several other types of STIs, including chlamydial infection, gonorrhea, herpes simplex virus type 2, and syphilis.”	Holmes K et al., 2004 [5]

## Materials and methods

Our analysis included an estimate of the current global gap in condom use and a cost-effectiveness analysis. We estimated current condom use and condom use required to meet all needs. Under three scale-up scenarios, we estimated the costs and impact associated with the triple protection that condoms afford to the prevention of unplanned pregnancies, HIV and STI transmission. We used these findings to estimate the cost-effectiveness of scaling up condom use and compare condom cost-effectiveness to related family planning and STI/HIV interventions.

This analysis covered the 75 ‘Countdown to 2015’ countries and an additional six UNAIDS Fast-Track to 2030 countries in S1 Table for a total of 81 countries. Fast-Track to 2030 countries (35 total) are those that adopted UNAIDS’ Fast Track Strategy to double the number of people on life-saving HIV treatment by 2020. The analysis covered the period from 2015 to 2030. Table 2 presents the three scenarios we modelled using different trends in condom use to consider the cost and impact of investment in condoms.

**Table 2:** Description of scenarios used in condom investment case.

Scenario	Description
**Low**	Condom use (for family planning, HIV and STI prevention) levels from 2015 are maintained and held constant from 2016–2030.
**Medium**	Trends in condom use (for family planning, HIV and STI prevention) are used to predict condom use from 2016–2030. For family planning, we used the United Nations Population Division’s projections of modern contraceptive prevalence through 2030. For HIV and STI prevention, we used the change in high-risk condom use rate between the two most recent surveys in each country to project annual increases through 2030 UN, 2015 [6]
**High**	Condom use is scaled up to meet all unmet need for family planning by 2020, and to 90% of sex acts at risk for HIV or other STI by 2020 (per updated Fast-Track country projections) UNAIDS, 2016 [7]

### Condom use

We defined the current gap in condom use as the difference between the current level of condom use and the total need. For condom use associated with family planning, we used data and sources detailed in S2 Table to estimate current condom use for family planning among women of reproductive age (i.e., 15–49 years) using the contraceptive prevalence rate for modern contraception among all women [8] and the proportion using condoms as their primary method of family planning [8–9]. These rates are multiplied by the number of women of reproductive age for each country [10] and summed to provide an estimate of the current number of couples using condoms for family planning in the 81 analysis countries. Numbers of users were converted to numbers of condoms by applying the Couple-Years of Protection (CYP) conversion factor for condoms of 120 condoms per user per year.

# of women of reproductive age x mCPR among all women x proportion of condom method mix among all women x 120 condoms per CYP

To estimate the total need for condoms for family planning, we assumed that condoms remain at the current proportion of the method mix, and that contraceptive prevalence increases to current use plus unmet need [8–9].

$$\begin{array}{l} \#\;of\;women\;of\;reproductive\;age\;x\;[mCPR\;among\;all\;women\\ +unmet\;need]\;x\;proportion\;of\;condom\;method\;mix\;among\;all\;women\;x\;120\;condoms\;per\;CYP \end{array}$$

Current level of condom use for HIV and STI prevention was estimated among population groups at risk for contracting HIV and STIs: female sex workers (FSW), men who have sex with men (MSM), transgenders, prisoners, those with multiple partners, and sero-discordant couples. Estimates of population sizes for these populations were from data submitted to UNAIDS in Spectrum files or through the Global AIDS Response Program Reporting system (GARPR) as part of national estimates prepared by programs in concentrated epidemics and data from Modes of Transmission studies in generalized epidemics. The proportion of the population with multiple partners is from Demographic and Health Survey (DHS) reports in generalized epidemics and Spectrum files for concentrated epidemics. The number of HIV sero-discordant couples is estimated from prevalence based on analysis of DHS data for 20 countries. Condom use rates are those reported in DHS and AIDS Indicator Surveys (AIS) among those who have multiple partners and those engaging in commercial sex, and special studies of condom use among key populations reported by country programs to UNAIDS through the GARPR. The global assumptions that we used for annual coital frequency are in Table 3 [11–14].

**Table 3:** Annual coital frequency assumptions among risk groups used to estimate condom use.

Risk Group	Annual Coital Frequency
Female sex workers	220 acts per year
Men who have sex with men	100 acts per year
Transgenders	100 acts per year
Prisoners	20 acts per year
Those with multiple partners	100 acts per year
Sero-discordant couples	70 acts per year

To estimate the total need for condoms for HIV and STI protection, we assumed 90% condom use among the risk groups. Detailed tables showing the calculation of the global condom gap by country, including population size estimates and condom use levels, are available in Tables A-E in S1 File.

### Addressing double and triple counting of condom use

Because a person can use a condom simultaneously for pregnancy, HIV, and STI prevention, it was important in our analysis to avoid potential double- or triple-counting of condom use. To minimize double- or triple-counting, we segmented the universe of condom users into risk groups in relation to HIV and STI transmission.

- We assumed that anyone who, based on DHS data, reports using a condom as their primary method of family planning is at low risk of HIV and STI transmission. We categorized condoms associated with these users as family planning condoms. For measuring impact, however, we assumed that a condom used for family planning also affords protection against HIV and STI transmission.
- We also identified condom users at medium and high risk of HIV and STI transmission. These users include FSWs, MSM, transgender males, sero-discordant couples, prisoners, and those with multiple partners. We categorized condoms associated with these users as HIV/STI condoms. For purposes of impact, we assumed that a condom used by anyone in these groups protects simultaneously against HIV and other STIs, but does not afford any protection against unintended pregnancy. Although this latter assumption likely underestimates the family planning benefit some high-risk condom users may receive, we preferred to be conservative to avoid any chance of double counting.

### Unit cost estimates

We calculated scale-up costs based on estimates of unit costs to provide condoms. We assigned FP condom users to one of two possible sources: clinical or social marketing. Family planning users who reported on the DHS having obtained condoms from a public source we assigned to the clinical source; those who obtained condoms from a private source we assigned to the social marketing source. We used Weissman’s (2014) [15] estimates of 2015 family planning costs by country as a proxy for clinical costs. These estimates include supplies, labor, and program costs. For social marketing unit costs we relied on PSI’s (2009) [16] estimates of gross cost per condom distributed by country, adjusted to 2015 prices. For countries where PSI lacked data, we used PSI’s average unit distribution cost ($0.18, adjusted for inflation). We assigned all HIV/STI condom users to the social marketing source, calculating unit costs in the same way we did for family planning social marketing users. We assumed unit cost for HIV/STI condom users to be the same regardless of the risk group [15]; these can be found in Table G in S1 File. We calculated all costs in constant 2015 $US and used a 3% discount rate. We also carried out sensitivity analysis using a 0% and 6% discount rate.

### Impact of condoms for family planning, and preventing HIV and STIs

#### Family planning impact

We estimated the impact of condoms in terms of birth averted and disability-adjusted Life years (DALYs) averted associated with the three scale-up scenarios. For births averted, we estimated the impact of changing rates of contraceptive prevalence on the number of births using the Bongaarts proximate determinants of fertility framework [17]. We started with the number of births in each country in 2015 [18]. The ratio of the contraceptive index (1 − 1.08 *x CPR x effectivness*) in each future year to the contraceptive index in the base year is applied to the base year Total Fertility Rate (TFR) to determine the TFR in each year. The coefficient 1.08 is an adjustment for the fact that some women who are infecund due to being post-menopausal will still be counted as contraceptive users if they are using a long term method such as sterilization. The number of births in each future year is determined from the TFR and the number of women of reproductive age. To calculate DALYs averted from use of condoms for contraception, we used country-specific coefficients from the Marie Stopes International IMPACT2 model, which represent the number of DALYs averted per condom. For our base-case calculation we considered DALYs averted only through maternal (not child) death or disability averted. We also carried out a sensitivity analysis, taking into account child DALYs averted. We did not discount the DALYs averted through condoms used for family planning.

#### Impact of condoms for HIV prevention

We estimated the impact of condoms HIV effectiveness in terms of infections and DALYs averted. To estimate the impact of condoms in preventing HIV infection we used the Goals model to simulate the HIV epidemic over time under the three scale-up scenarios. The Goals model is a mathematical simulation model that estimates the spread of HIV over time based on the size of each population risk group and the behaviors in each group, and is part of the Spectrum software package (available at www.avenirhealth.org/). The model is first fit to data on historical trends and then used to project the future epidemic. We set up the model for 39 countries with the largest HIV epidemics. For countries for which we did not have a Goals model application, we used the official estimate of new infections in 2015 from UNAIDS and specified the trend from 2015 to 2030 on the basis of one of the Goals countries selected as the best proxy (based on location and similar epidemic type). The model projects the number of new HIV infections, AIDS deaths, and DALYs under each scenario. The assumptions used to estimate the effectiveness of condoms for HIV prevention can be found in S4 and S5 Tables. We did not discount the DALYs averted from condom use for HIV prevention.

#### Impact of condoms for STI prevention

We estimated the impact of condoms in terms of sexually transmitted infections averted and DALYs averted for four common STIs: syphilis, gonorrhea, chlamydia, and HSV-2. First, we drew on published systematic reviews and meta-analyses of controlled longitudinal studies to identify use-effectiveness of condoms against STIs. These estimates generally expressed effectiveness as a reduction in the risk of STI transmission (relative risk or odds ratio) for people using condoms, over a given but usually unspecified time period, and are presented in S6 Table. Using these condom effectiveness estimates to represent the risk over a year, we applied these to estimates of annual STI cases—without conversion or adjustment for differing time periods or number of sex acts in the different studies. We used WHO’s latest global and regional STI burden data to estimate the baseline burden of STIs avertable by condoms for syphilis, gonorrhea, and chlamydia [19]. For HSV-2, we drew on another recent global and regional level estimation [20] to calculate the baseline burden. From the regional burden estimates, we extrapolated to corresponding country estimates by applying the regional incidence rates, presented in S7 Table, to all countries in the region, for men and women separately. Next, we estimated the number of infections averted per condom used by dividing the number of infections averted from baseline condom use by the total number of condoms used at baseline in the appropriate risk groups. We multiplied the infections averted per condom used by the number of condoms used in each scenario to generate estimates of infections averted. Finally, we converted STI infections averted to DALYs averted using an average DALY per incident STI episode, calculated using DALY estimates from the 2013 Global Burden of Disease [21], which were built on the same WHO 2012/2015 STI burden estimates, and are shown in S8 Table. We did not discount the DALYs averted from condom use for STI prevention.

For the medium and high scenarios, we calculated an incremental cost-effectiveness ratio in terms of cost per DALY averted. The DALY averted measure standardizes health impact across disease or health areas, and thus is well suited to capture the combined effect of condom use on prevention of pregnancy, and of HIV and STI transmission including morbidity as well as mortality impacts.

### Sensitivity analysis

The cost-effectiveness analysis builds on assumptions about key parameters, many of which have significant associated uncertainty. Sensitivity analysis helps determine the extent to which changes in these assumptions might substantially alter the findings. Parameters that hold a significant degree of uncertainty include unit cost to distribute condoms, coital frequency for specific HIV/STI risk groups and for couples using condoms for FP, and the discount rate used to value costs. We also examined the extent to which our findings might vary when including estimates of child DALYs averted to our FP impact estimates. The sensitivity range for each of these parameters are presented in S9 Table. We carried out one-way and multi-way sensitivity analyses on these parameters.

## Results

### Global condom gap

**Fig 1:**
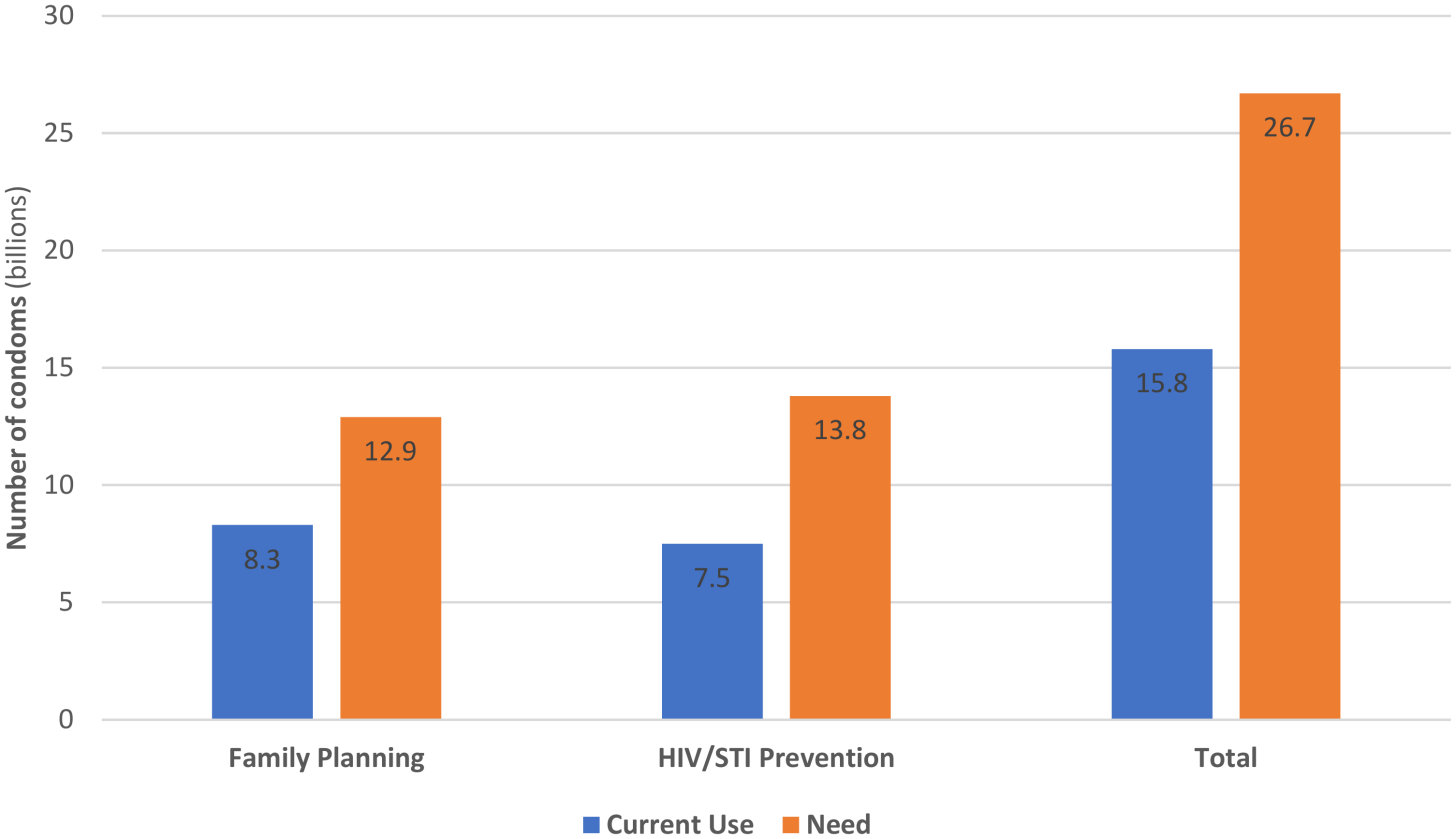
Global current use and need for condoms for family planning and STI/HIV prevention, 2015 (billions), 81 countries.

Our estimates show current use of 15.8 billion condoms in 2015 (8.3 billion condoms for family planning condoms and 7.5 billion condoms for HIV/STI prevention). We estimate the total need for condoms at 26.7 billion (12.9 billion for FP and 13.8 billion for HIV/STI prevention); this leaves a gap of 10.9 billion condoms (Fig 1).

Table 4 reveals the family planning condom gap is concentrated in Southern Asia, and Middle and Western Africa.

**Table 4:** Global estimates of current use, need, and gap in condoms for family planning, by region, 2015 (millions).

	# of women of reproductive age	Current use of condoms for family planning	Current need for condoms for family planning	Gap in condoms for family planning
**AFRICA**				
Eastern Africa	93	235	492	257
Middle Africa	34	241	961	720
Northern Africa	42	21	28	7
Southern Africa	17	152	191	39
Western Africa	81	332	971	639
**ASIA**^a^				
Central Asia	59	680	1,064	384
Eastern Asia	370	2,113	2,288	175
Southern Asia	445	2,664	4,445	1,781
South-Eastern Asia	142	374	573	199
Western Asia	42	89	152	63
**LATIN AMERICA AND THE CARIBBEAN**				
Caribbean	4	32	68	36
Central America	39	157	202	45
South America	68	564	714	150
**OCEANIA**				
Melanesia/Micronesia/Polynesia	2	3	9	6
**NORTH AMERICA**				
	73	646	775	129
**GRAND TOTAL**	**1,512**	**8,303**	**12,933**	**4,630**

^a^ In the Asia region, Central Asia includes the Russian Federation (with 35 million women of reproductive age (WRA)), Eastern Asia includes China (WRA = 363 million), Southern Asia includes India (337 million), and Southeastern Asia includes Indonesia (WRA = 70 million).

The condom gap for HIV and STI prevention is concentrated in the multiple partner group as seen in Table 5. Detailed tables, including global estimates of current use, need and gap for condoms by country can be found in Tables A-C, F and I in S1 File.

**Table 5:** Global estimates of current use, need, and gap in condoms for HIV and STI prevention, among risk groups (2015) in millions of condoms.

Risk group	Current use of condoms for HIV and STI prevention	Current need for condoms for HIV and STI prevention	Gap in condoms for HIV and STI prevention
**Sex workers**	2,500	2,900	400
**Men who have sex with men**	1,100	1,600	500
**Transgenders**	100	100	10
**Prisoners**	100	200	60
**Multiple partners**	3,500	8,300	4,800
**Sero-discordant couples**	300	700	400
**TOTAL**	7,500	13,800	6,170

### Cost to provide condoms

From 2015–2030, the total cost of condoms varies from $59.6 billion in the Baseline Scenario to $61.5 billion under the Medium Scenario to $87.2 billion under the High Scenario as seen in Table 6. Additional details by country are in Tables J-L in S1 File.

**Table 6:** Total cost of condoms for FP and HIV/STI prevention, by scenario, in billions $US, 2015–2030, 81 countries.

	Baseline Scenario	Medium Scenario	High Scenario
	(2015–2030)		
**Family Planning**	$25.2	$26.3	$30.3
**HIV and STI Prevention**	$34.5	$35.2	$56.9
Sex workers	$14.6	$14.8	$15.7
Men who have sex with men	$5.8	$5.9	$7.5
Transgenders	$0.5	$0.5	$0.5
Prisoners	$12.2	$12.5	$30.1
Multiple partners	$0.9	$1.0	$2.4
Sero-discordant couples	$0.5	$0.5	$0.7
**TOTAL**	**$59.6**	**$61.5**	**$87.2**

From 2015 to 2030, the total incremental cost of condoms is $1.9 billion for the Medium and $27.6 billion for the High Scenarios, as presented in Table 7 and in greater detail in Table M in S1 File. During this period, Africa accounts for almost 90% of incremental costs under the Medium Scenario and 50% of costs under the High Scenario. Asia also contributes significantly to incremental costs under the High Scenario, accounting for 38% of the total.

**Table 7:** Incremental cost of condoms for FP and HIV/STI prevention, in millions, US$, 2015–2020, 2015–30, total and by region.

	Medium vs Low Scenario	High vs Low Scenario	Medium vs Low Scenario^a^	High vs Low Scenario
	(2015–2020)		(2015–2030)	
**Family Planning**	$141	$704	$1,179	$5,172
**HIV and STI Prevention**	$95	$8,056	$718	$22,456
**Total**	$236	$8,760	$1,896	$27,628
**By Region**				
**Africa**	$208	$4,083	$1,687	$13,839
Eastern Africa	$57	$1,313	$437	$4,136
Middle Africa	$75	$1,026	$623	$3,770
Northern Africa	$0	$175	$2	$512
Southern Africa	$1	$79	$8	$238
Western Africa	$75	$1,490	$617	$5,183
**Asia**	$10	$3,614	$92	$10,588
Central Asia	$6	$218	$36	$724
Eastern Asia	-$35	$2,491	-$204	$6,604
Southern Asia	$35	$388	$233	$1,674
South-Eastern Asia	$3	$401	$19	$1,227
Western Asia	$1	$116	$9	$360
**Latin America and the Caribbean**	$18	$668	$117	$1,996
Caribbean	$17	$91	$110	$305
Central America	$0	$250	$2	$720
South America	$1	$328	$5	$971
**Oceania**	$2	$100	$110	$305
Melanesia/ Micronesia/ Polynesia	$2	$100	$110	$305
**North America**	-$2	$295	-$13	$856
North America	-$2	$295	-$13	$856
**TOTAL**	**$235**	**$8,760**	**$1,896**	**$27,627**

^a^ The incremental costs in the medium scenario are negative for some regions because the UN projects CPR for certain countries (i.e., China, Vietnam, Brazil, Jamaica, and the U.S.) to fall between 2015–2030.

### Impact of triple protection from condoms

#### Impact of condoms for FP

As shown in Table 8, from 2015 to 2030 under the Medium Scenario condoms for family planning avert 97 million births and 1.7 million DALYs, while under the High Scenario, condoms avert 419 million births and 5.0 million DALYS.

**Table 8:** Condom effectiveness: Births averted and DALYs averted, by region (2015–2030).

	Total Births Baseline Scenario (2015–2030)(millions)	Births Averted(2015–2030)(millions)		FP DALYs Averted (2015–2030)	
		Low-Medium	Low-High	Low-Medium	Low-High
**AFRICA**					
Eastern Africa	375	39	80	235,044	437,371
Middle Africa	175	12	40	491,584	1,725,770
Northern Africa	82	5	15	1,138	5,178
Southern Africa	19	0.7	3	7,361	29,421
Western Africa	381	28	62	727,423	1,566,633
**ASIA**					
Central Asia	46	2	10	2,774	15,803
Eastern Asia	190	(16)	29	(4,118)	10,534
Southern Asia	517	21	96	227,377	1,000,742
South-Eastern Asia	151	3	25	5,659	51,211
Western Asia	69	3	17	2,644	13,054
**LATIN AMERICA AND THE CARIBBEAN**					
Caribbean	4	0.2	1	7,050	34,332
Central America	39	2	10	518	7,584
South America	52	(0.7)	14	206	36,962
**OCEANIA**					
Melanesia/ Micronesia/ Polynesia	4	0.1	0.9	403	2,900
**NORTH AMERICA**					
North America	73	(2)	16	(2,174)	19,082
**Total**	**2,175**	**97**	**419**	**1,702,889**	**4,956,578**

#### Effectiveness of condoms against HIV

From 2015 to 2030, under the Medium Scenario condoms prevent 0.4 million new HIV infections and avert 3.4 million DALYs. During this same period, under the High Scenario, condoms prevent 16.8 million new infections and avert 204.6 million DALYs as seen in Table 9.

**Table 9:** Condom effectiveness: HIV infections and DALYs averted, by region (2015–2030).

	2015–2030			
	HIV Infections Averted		DALYs Averted	
	Low-Medium	Low-High	Low-Medium	Low-High
**AFRICA**				
Eastern Africa	183,000	5,431,000	1,622,000	60,868,000
Middle Africa	95,000	933,000	834,000	12,412,000
Northern Africa	2,000	161,000	14,000	1,770,000
Southern Africa	12,000	3,171,000	102,000	40,960,000
Western Africa	60,000	2,000,000	554,000	26,153,000
**ASIA**				
Central Asia	-^a^	1,328, 000	0	14,802, 000
Eastern Asia	-^a^	191, 000	0	2,515, 000
Southern Asia	10, 000	861, 000	76, 000	11,176, 000
South-Eastern Asia	3, 000	1,200, 000	29, 000	16,085, 000
Western Asia	5, 000	316, 000	32, 000	3,550, 000
**LATIN AMERICA AND THE CARIBBEAN**				
Caribbean	4, 000	16, 000	40, 000	224, 000
Central America	200	45, 000	2, 000	613, 000
South America	14, 000	688, 000	98, 000	8,121, 000
**OCEANIA**				
Melanesia/ Micronesia/ Polynesia	60	25, 000	500	328, 000
**NORTH AMERICA**				
North America	0	456,000	0	5,039,000
**TOTAL**	**388,000**	**16,823,000**	**3,404,000**	**204,617,000**

^a^ Some regions have no infections averted in the medium scenario. This occurs when there is no evidence of an increasing trend in condom use rates between the last two national surveys. In those cases condom use rates in the medium scenario are identical to those in the Low scenario.

#### Effectiveness of condoms against STIs

Table 10 shows our estimates of baseline STI incidence cases of gonorrhea, chlamydia, syphilis, and HSV-2 of 103 million among men and 93 million among women. Under the Medium Scenario, condom use averts 16.5 million infections and 1.1 million DALYs, while under the High Scenario condom use averts 733.7 million infections and 30.1 million DALYs. Additional details for each country are presented in Table N in S1 File.

**Table 10:** Condom effectiveness: STI infections and DALYs averted, by region (2015–2030), medium and high scenarios.

	2015		2015–2030			
	Total Baseline STI incident cases		Total STI infections averted		Total DALYs averted from STIs	
	Men	Women	Medium Scenario	High Scenario	Medium Scenario	High Scenario
**AFRICA**						
Eastern Africa	5,599,000	8,191,000	9,633,000	76,875,000	750,000	6,117,000
Middle Africa	2,106,000	3,079,000	3,863,000	27,711,000	283,000	2,101,000
Northern Africa	2,227,000	2,080,000	200	23,801,000	0	1,050,000
Southern Africa	1,067,000	1,502,000	7,000	5,314,000	100	375,000
Western Africa	5,053,000	7,260,000	935,000	72,725,000	59,000	5,855,000
**ASIA**						
Central Asia	2,006,000	1,912,000	7,000	7,853,000	100	316,000
Eastern Asia	44,800,000	34,640,000	(12,000)	191,496,000	(200)	2,966,000
Southern Asia	16,622,000	14,272,000	68,000	149,852,000	1,000	6,101,000
South-Eastern Asia	9,472,000	8,081,000	1,054,000	83,068,000	16,000	1,863,000
Western Asia	2,136,000	2,028,000	1,000	18,727,000	20	803,000
**LATIN AMERICA AND THE CARIBBEAN**						
Caribbean	217,000	366,000	737,000	1,780,000	25,000	61,000
Central America	2,372,000	3,942,000	600	13,662,000	10	454,000
South America	4,169,000	6,894,000	163,000	27,294,000	6,000	925,000
**OCEANIA**						
Melanesia/ Micronesia/ Polynesia	248,000	196,000	200	1,950,000	0	31,000
**NORTH AMERICA**						
North America	4,511,000	7,728,000	(5,000)	31,593,000	(80)	1,067,000
**TOTAL**	**102,605,000**	**93,098,000**	**16,450,000**	**733,701,000**	**1,140,000**	**30,086,000**

### Cost-effectiveness

#### Cost per DALY averted

Table 11 displays the incremental cost-effectiveness ratio (ICER). For the 2015–30 Medium Scenario it is $304 per DALY averted and for the High Scenario it is $115 per DALY averted. Additional country and regional details are in Table P in S1 File.

**Table 11:** Incremental cost, effectiveness, and cost-effectiveness ratio of condoms, (2015–2030).

	Scenario (2015–2030)	
	Medium over Low	High over Low
**Incremental Cost ($ millions)**	$1,896	$27,627
**Incremental Effectiveness (DALYs averted), millions**	6.2	239.7
**Incremental Cost-Effectiveness Ratio (ICER)**	$304	$115

#### Comparison to cost-effectiveness thresholds

Following the recommendations of the Commission on Macroeconomics and Health, WHO-CHOICE sets standards for cost-effectiveness across health interventions in terms of cost per DALY averted, classifying interventions as:

- Highly cost effective (if the ICER is less than one GDP per capita),
- Cost effective (if the ICER is between one and three times GDP per capita), or
- Not cost effective (if the ICER is higher than 3 times GDP per capita).

As seen in Table 12, the ICER for condom investment is far below the weighted annual GDP per capita of $7,005 for the 81 investment case countries, placing it in the category of highly cost-effective. The ICER by region produces similar results, and a country-by-country analysis shows that condom investment for 2015–2030 is highly cost-effective or cost-effective in 71 of 81 countries under the Medium Scenario, and 79 of 81 countries under the High Scenario. These additional analyses can be found in Tables P and Q in S1 File.

**Table 12:** Condom cost-effectiveness, on average for all condom investment framework countries, 2015–2030.

Scenario	ICER	GDP Per Capita	% of annual GDP per capita	Rating
**Medium over low**	$304	$7,005	4%	Highly cost-effective
**High over low**	$115	$7,005	2%	Highly cost-effective

### Sensitivity analysis

For the Medium Scenario, adding family planning child DALYs to the impact calculation greatly reduces the ICER, to $65 from the base case value of $304 as seen in Table 13. Changes in the other parameters also produce large shifts in the ICERs. We also carried out a multi-way sensitivity analysis that combined all the parameters to produce a single value associated with setting all parameters at the low end of the sensitivity range and another value for setting all parameters at the high end of the sensitivity range. This produces an ICER range of between $33 and $539. In the High Scenario, changing the unit cost assumption has the greatest influence on ICER. Changes in the other parameters also produce shifts in the ICERs. The multi-way sensitivity analysis produces a range of ICER of between $43 and $212.

**Table 13:** Range of ICERs resulting from the sensitivity analysis, medium and high scenarios, 2015–2030.

	**Medium Scenario—Parameter range for sensitivity analysis**		
**Parameter**	**Low**	**Base**	**High**
**One-way sensitivity**			
** FP Child DALYs**	$304	$304	$65
** Discount rate for costs for FP and HIV/STI prevention**	$414	$304	$227
** Unit Cost**	$228	$304	$379
** Coital frequency**	$288	$304	$316
**All-way sensitivity**	$33	$304	$539
	**High Scenario—Parameter range for sensitivity analysis**		
**Parameter**	**Low**	**Base**	**High**
**One-way sensitivity**			
** FP Child DALYs**	$86	$115	$144
** Discount rate for costs**	$148	$115	$92
** Unit Cost**	$93	$115	$132
** Coital frequency**	$115	$115	$91
**All-way sensitivity**	$43	$115	$212

### Comparison of condoms to other interventions aimed at HIV/STI/pregnancy prevention

**Fig 2:**
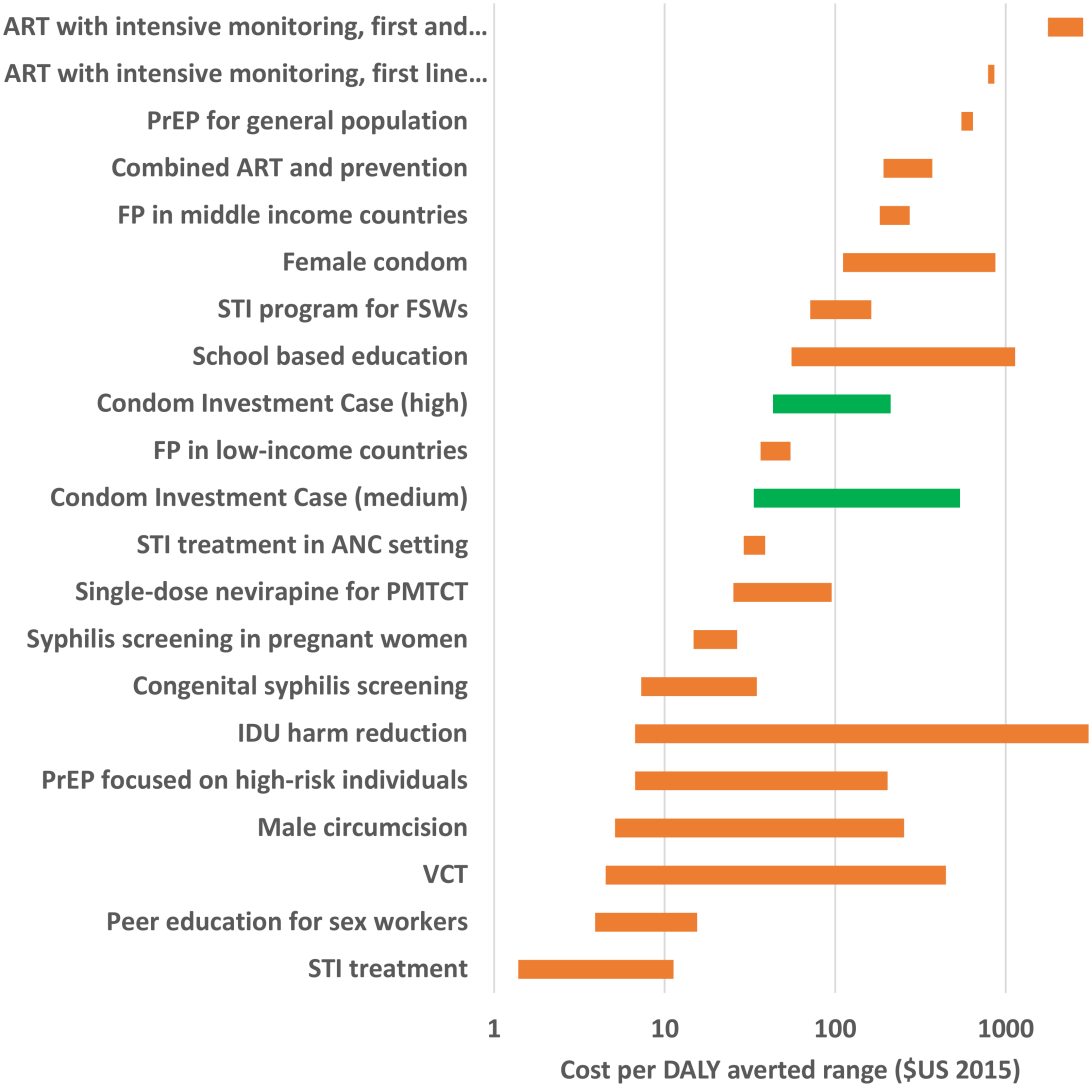
Cost-effectiveness of various HIV/STI/FP interventions, cost per DALY averted, $US 2015.

The analysis in the previous section showed that, according to the WHO thresholds, the condom investment is a highly cost-effective intervention. But, how do condoms compare to other interventions with similar aims? To answer that question, we examined published data on cost-effectiveness of a range of STI/HIV/pregnancy prevention interventions (Fig 2), identifying any reported cost effectiveness ratios in cost per DALY averted (or a value that could be converted to DALY averted). Most of these are HIV interventions, with fewer focused on STIs and just a few on family planning. They reveal the cost per DALY averted converted to $US 2015 on a logarithmic scale for various interventions, including the High and Medium Scenarios in the condom investment case (shown as the green bars on the figure). The sources for the studies used to generate Fig 2 can be found in Table R in S1 File. The width of the bar represents the range between the low and high cost-effectiveness ratio, in cases where the study reported a range of results. The low-end results for the condom investment case ICERs are roughly comparable to other prevention interventions. At the higher end of our sensitivity analyses, the condom investment case ICERs compare less favorably to other interventions. Our low-end estimates are also similar to the ICERs for a range of other reproductive and maternal and child health interventions recently analyzed in a forthcoming study (Horton and Levin forthcoming).

## Discussion

With health funding levels stagnant or falling, it is important to understand the cost and health impact associated with prevention technologies. This study is one of the first to try to quantify the cost and combined health impact of condom use as a means to prevent unwanted pregnancy, and to prevent transmission of STIs, including HIV. We found an annual gap between current and desired use of 10.9 billion condoms—4.6 billion for family planning and 6.3 billion for HIV and STIs. The UNAIDS 2016–2021 Strategy sets a global target of increasing the availability of condoms to 20 billion per year by 2020 in low- and middle-income countries, and achieving 90% condom use during the most recent sexual activity with a non-regular partner. Under a high scenario that completely reduces that gap between current and desired use of 10.9 billion condoms, we found that by 2020 countries could avert 47 million births, 4.5 million HIV infections, and 231 million other STI infections. These translate into 22.3 million DALYs averted. Over the long term, eliminating the gap could allow countries to avert 240 million DALYs by 2030. The health gains are more modest under a medium scenario that assumes the continuation of current use trends.

The additional cost in the 81 countries through 2020 is $236 million under the medium scenario and $8.8 billion under the high scenario. Through 2030, the cost is $1.9 billion under the medium and $27.6 billion for the high scenario. Through 2020, we found an incremental cost of $534 per DALY averted for the medium and $394 for the high scenario. Through 2030, these cost-effectiveness ratios fall substantially, to $304 for the medium and $115 for the high scenario. For all time frames and scenarios, these ratios are highly cost-effective per WHO CHOICE standards. Moreover, when placed against other interventions with similar sexual and reproductive health aims, condom investments compare favorably.

These findings should be viewed in light of some important methodological limitations based on the study’s underlying assumptions. First, our analysis uses a narrow definition of condom use. To determine condom use for family planning, we use DHS data which refers to women who report using condoms as their main method of family planning. For HIV/STI prevention, we rely on reports of condom use at last sex by men and women who report having more than one partner in the last year. We do not have information about correct or consistent use of condoms. Our analysis also may not include condom use for family planning by key population groups. Second, we began our analysis by estimating the number of condoms used in 2015 for family planning, but we did not calculate the actual number of condoms distributed in 2014 (the year prior to the baseline year in this analysis) to compare these to. We did attempt to estimate the availability of condoms from the private sector and public sector in 2015, as seen in Table K in S1 File. The estimate of 8.3 billion condoms currently used in 2015 for family planning is based on DHS reporting of the use of condoms for family planning. It is estimated that of these 8.3 billion condoms, 6.1 billion were from the private sector (and 2.2 billion from the public sector or from condoms that were socially marketed). Third, we were conservative in our estimates of condom use and impact, in part to minimize double-and triple- counting of condom. For example, we assumed no births averted impact from the condoms used by groups at medium and high risk of HIV and STI infection; some limited impact likely exists. For lack of information on condom prevention effectiveness and case incidence rates from other STIs (e.g., trichomoniasis and HPV-2), we measured impact of condom use on only four common STIs. We also assumed that condom use among low-risk groups would affect probability of transmission of only one of the four STIs. In addition, in our tally of family planning condoms, we did not count condoms that women reported using in addition to a more effective contraceptive method such as oral pill or injectable. Thus, our impact estimates likely understate the true impact of condoms on prevention. Fourth, there was substantial uncertainty around certain key parameters including coital frequency, discount rate for costs, and unit cost to provide condoms. We addressed this through sensitivity analysis. Fifth, our base case did not count the impact of condoms on child DALYs averted from family planning use. When the impact of child DALYs averted from family planning use are incorporated into the analysis, investments in condoms are much more cost-effective. Any comparisons to previous cost-effectiveness studies measuring contraceptive impact, which generally include impact on child DALYs, should consider this difference. Finally, care should be taken comparing our results with other studies. Other studies might calculate both cost and effectiveness differently. Moreover, differences in costs between countries further hamper comparisons. We in part addressed these shortcoming by drawing from a broad and extensive range of other studies against which to compare the condom investment results.

With these limitations in mind, our results point to a high potential impact of male condoms, and thus the value of continued investment in them. Meeting all demand for condom use would have a large health impact through prevention of unwanted pregnancy, and prevention of HIV and other STIs. Donors and country governments should expand programs that promote and distribute condoms, in line with the recent the recent Joint United Nations Programme on HIV/AIDS (UNAIDS) statement calling for “increased investments by donors and governments for the promotion and distribution of male (and female) condoms in order to ensure everyone has access to condoms to protect themselves and their partners from HIV, STIs and unintended pregnancies [6].”

## Supporting information

S1 File (XLSX)

S1 Table (PDF)

S2 Table (PDF)

S3 Table (PDF)

S4 Table (PDF)

S5 Table (PDF)

S6 Table (PDF)

S7 Table (PDF)

S8 Table (PDF)
