# Supplementary material for: The case for investing in the male condom
Source: PLoS One. 2017 May 16;12(5):e0177108. doi: 10.1371/journal.pone.0177108 (PMC5433691; doi:10.1371/journal.pone.0177108)
Supplement: S1 Table — (PDF) [file pone.0177108.s002.pdf]

S1 Table. List of countries included in condom investment analysis

CONDOM INVESTMENT CASE COUNTRIES	COUNTDOWN TO 2015 COUNTRY	UNAIDS FAST TRACK COUNTRY	TRACK20 COUNTRY[a]
**AFRICA**			
**EASTERN AFRICA**			
BURUNDI	X		X
COMOROS	X		X
DJIBOUTI	X		X
ERITREA	X		X
ETHIOPIA	X	X	X
KENYA	X	X	X
MADAGASCAR	X		X
MALAWI	X	X	X
MOZAMBIQUE	X	X	X
RWANDA	X		X
SOMALIA	X		X
SOUTH SUDAN	X	X	X
UGANDA	X	X	X
UNITED REPUBLIC OF TANZANIA	X	X	X
ZAMBIA	X	X	X
ZIMBABWE	X	X	X
**MIDDLE AFRICA**			
ANGOLA	X	X	
CAMEROON	X	X	X
CENTRAL AFRICAN REPUBLIC	X		X
CHAD	X	X	X
CONGO	X		X
DEMOCRATIC REPUBLIC OF CONGO	X	X	X
EQUATORIAL GUINEA	X		
GABON	X		
SAO TOME AND PRINCIPE	X		X
**NORTHERN AFRICA**			
EGYPT	X		X
MOROCCO	X		
SUDAN	X		X
**SOUTHERN AFRICA**			
BOTSWANA	X	X	
LESOTHO	X	X	X
NAMIBIA		X	
SOUTH AFRICA	X	X	X
SWAZILAND	X	X	
**WESTERN AFRICA**			
BENIN	X		X
BURKINA FASO	X		X
CÔTE D'IVOIRE	X	X	X
GAMBIA	X		X
GHANA	X	X	X
GUINEA	X		X
GUINEA-BISSAU	X		X
LIBERIA	X		X
MALI	X	X	X
MAURITANIA	X		X
NIGER	X		X
NIGERIA	X	X	X

SENEGAL	X		X
SIERRA LEONE	X		X
TOGO	X		X
**ASIA**			
**CENTRAL ASIA**			
KYRGYZSTAN	X		X
TAJIKISTAN	X		X
TURKMENISTAN	X		
UZBEKISTAN	X		X
UKRAINE		X	
RUSSIAN FEDERATION		X	
**EASTERN ASIA**			
CHINA	X	X	
DEMOCRATIC PEOPLE'S REPUBLIC OF KOREA	X		X
**SOUTHERN ASIA**			
AFGHANISTAN	X		X
BANGLADESH	X		X
INDIA	X	X	X
NEPAL	X		X
PAKISTAN	X	X	X
**SOUTH-EASTERN ASIA**			
CAMBODIA	X		X
INDONESIA	X	X	X
LAO PDR	X		X
MYANMAR	X	X	X
PHILIPPINES	X		X
VIET NAM	X	X	X
**WESTERN ASIA**			
AZERBAIJAN	X		
IRAN (ISLAMIC REPUBLIC OF)		X	
IRAQ	X		X
YEMEN	X		X
**LATIN AMERICA AND THE CARIBBEAN**			
**CARIBBEAN**			
HAITI	X	X	X
JAMAICA		X	
**CENTRAL AMERICA**			
GUATEMALA	X		
MEXICO	X		
**SOUTH AMERICA**			
BOLIVIA (PLURINATIONAL STATE OF)	X		
BRAZIL	X	X	
PERU	X		
**OCEANIA**			
**MELANESIA/MICRONESIA/POLYNESIA**			
PAPUA NEW GUINEA	X		X
SOLOMON ISLANDS	X		X
**NORTH AMERICA**			
UNITED STATES OF AMERICA		X	

[a] Track20 Countries (www.track20.org/) are included in this table to show from where the indicators pertaining to family planning use (mCPR, method mix, etc.) were used that related to all women of reproductive age.
