# Supplementary material for: The case for investing in the male condom
Source: PLoS One. 2017 May 16;12(5):e0177108. doi: 10.1371/journal.pone.0177108 (PMC5433691; doi:10.1371/journal.pone.0177108)
Supplement: S2 Table — (PDF) [file pone.0177108.s003.pdf]

S2 Table. Data and sources to estimate current condom use and condoms needed for family planning

Indicator	Source	Notes
Number of women of reproductive age	United Nations, Department of Economic and Social Affairs, Population Division (2015). World Population Prospects: The 2015 Revision, custom data acquired via website Nov 15, 2015.	
Contraceptive prevalence rate, modern method(mCPR, all women)	Track20[a], Avenir Health (analysis); DHS (data) - www.track20.org	For investment case countries that are also Track20 countries, we obtained the 2015 modeled mCPR which calculates mCPR using Track20's FPET Tool. The FPET Tool includes a country's historic and recent DHS, MICS, PMA2020 and other national survey data to estimate mCPR among married women and then convert these values to all women of reproductive age.
	Angola: average mCPR ratio of Democratic Republic of Congo and Zambia<br>***Equatorial Guinea:*** mCPR ratio from 2011 DHS<br>***Gabon:*** mCPR ratio from 2012 DHS<br>***Morocco:*** mCPR ratio for Mauritania<br>***Botswana:*** average mCPR ratio from Zimbabwe, Zambia, Angola and South Africa<br>***Namibia:*** mCPR ratio from 2013 DHS<br>***Swaziland:*** mCPR ratio from 2006 DHS<br>***Turkmenistan:*** mCPR ratio from 2000 DHS<br>***China:*** average mCPR ratios from DR Korea, Vietnam, Lao PDR, Myanmar, and Nepal<br>***Azerbaijan:*** mCPR ratio from 2006 DHS<br>***Guatemala:*** mCPR ratio from 2014-15 DHS<br>***Mexico:*** average of mCPR ratios from Guatemala, Honduras and Nicaragua<br>***Brazil:*** mCPR ratio from 1996 DHS<br>***Peru:*** mCPR ratio from 2014 DHS<br>***Iran (Islamic Republic of):*** average of mCPR ratios from Iraq, Afghanistan, Pakistan, Turkmenistan and Azerbaijan	For non-Track20 countries, we determined an adjusted 2015 mCPR for all women using either the ratio of mCPR among married women and mCPR among all women (for countries with these indicators in their DHS) or used the ratio of mCPR among married women and mCPR among all women from the region or a neighboring country

	Jamaica: mCPR ratio from Haiti<br>***Russian Federation:*** average of mCPR ratios from Mongolia and Ukraine<br>***Ukraine:*** mCPR ratio from 2007 DHS<br>***United States of America:*** mCPR ratio from National Health Statistics Report No 60. October 18, 2012 Table 3.	
Percent of total family planning users using condoms	Track20, Avenir Health (analysis); DHS (data) - www.track20.org<br>and<br>United Nations, Department of Economic and Social Affairs, Population Division (2015). Model-based Estimates and Projections of Family Planning Indicators 2015. New York: United Nations.	Track20 calculates method mix by dividing the method specific prevalence rate by the total modern contraceptive prevalence rate. Method mix data for each country were obtained from the most recent DHS, MICS, PMA2020 or national cross-sectional survey report. For additional details please refer to: http://www.track20.org/pages/data/data_sources_and_methodology<br>For non-Track20 countries, used method mix from UN 2015 Population Prospects
Percentage of women with an unmet need for modern methods of contraception, among women married/in union	Track20, Avenir Health (analysis); DHS (data) - www.track20.org	For investment case countries that are also Track20 countries, we obtained the 2015 modeled percentage of unmet need for modern methods from the Track20 website. This modeled percentage is calculated using the FPET Tool.
	United Nations, Department of Economic and Social Affairs, Population Division (2015). Model-based Estimates and Projections of Family Planning Indicators 2015. New York: United Nations.	For those investment case countries that are not Track20 countries, we obtained the 2015 modeled percentage of women married/in union with an unmet need for modern methods from the UN Model-based Estimates and Projections.

[a] The Track20 Project monitors progress towards achieving the goals of the global FP2020 initiative (an additional 120 million modern method users between 2012 and 2020 in the world's 69 poorest countries). Track20 works directly with governments in participating FP2020 countries to collect, analyze and use data to monitor progress annually in family planning and to actively use data to improve family planning strategies and plans. More information at: www.track20.org/
