# Supplementary material for: The case for investing in the male condom
Source: PLoS One. 2017 May 16;12(5):e0177108. doi: 10.1371/journal.pone.0177108 (PMC5433691; doi:10.1371/journal.pone.0177108)
Supplement: S3 Table — (PDF) [file pone.0177108.s004.pdf]

S4 Table. Investment case countries (with Goals Models and proxy assigned as appropriate)

Goals Model Countries	Investment Case Countries
Bangladesh	
Botswana	
Brazil	Bolivia (Plurinational State of)
Burkina Faso	Benin, Cote d'Ivoire, Equatorial Guinea, Guinea, Guinea-Bissau, Mali
Cambodia	Lao People's Democratic Republic
Cameroon	Central African Republic, Chad, Gabon, Sao Tome and Principe, Togo
China	Democratic People's Republic of Korea
Democratic Republic of the Congo	Congo
Ethiopia	Djibouti, Eritrea, Somalia
Ghana	Gambia
India	Comoro, Madagascar, Myanmar, Nepal
Indonesia	Papua New Guinea, Philippines, Solomon Islands
Kenya	
Lesotho	
Liberia	
Malawi	
Mexico	Guatemala, Haiti, Jamaica, United States of America
Mozambique	
Namibia	Angola
Nigeria	
Pakistan	Iran (Islamic Republic of), Iraq, Afghanistan, Morocco, Sudan
Russian Federation	
Sierra Leone	
South Africa	
Swaziland	
Uganda	Rwanda, South Sudan
Ukraine	Egypt, Azerbaijan, Kyrgyzstan, Tajikistan, Turkmenistan
United Republic of Tanzania	Burundi
Viet Nam	
Zambia	
Zimbabwe	
