# Supplementary material for: The case for investing in the male condom
Source: PLoS One. 2017 May 16;12(5):e0177108. doi: 10.1371/journal.pone.0177108 (PMC5433691; doi:10.1371/journal.pone.0177108)
Supplement: S4 Table — (PDF) [file pone.0177108.s005.pdf]

S5 Table. New HIV infections in 2015 by country

Country	New HIV infections	Country	New HIV infections
AMRO		EURO	
**Bolivia (Plurinational State of)**	1,254	**Azerbaijan**	892
**Brazil**	44,657	**Kyrgystan**	872
**Guatemala**	2,798	**Tajikistan**	1,882
**Haiti**	6,850	**Turkmenistan**	822
**Jamaica**	1,431	**Ukraine**	14,118
**Mexico**	7,345	**Uzbekistan**	407
**Peru**	2,217	**Russian Federation**	111,560
**United States of America**	47,312[c]		
SEARO		WPRO	
**Bangladesh**	981	**Cambodia**	814
**Democratic People's Republic of Korea**	152	**China**	60,339
**India**	85,524[b]	**Lao People's Democratic Republic**	897
**Indonesia**	71,569	**Papua New Guinea**	1,921
**Myanmar**	7,080	**Philippines**	7,188
**Nepal**	1,155	**Solomon Islands**	30
		**Vietnam**	14,701
EMRO			
**Afghanistan**	1,039	**Morocco**	2,037
**Djibouti**	580	**Pakistan**	22,743
**Egypt**	1,360	**Somalia**	3,926
**Iran (Islamic Republic of)**	7,373	**Sudan**	5,280
**Iraq**	9,236	**Yemen**	778
AFRO			
**Angola**	27,393	**Liberia**	1,365
**Benin**	3,028	**Madagascar**	2,664
**Botswana**	9,199	**Malawi**	40,826
**Burkina Faso**	4,867	**Mali**	10,054
**Burundi**	859	**Mauritania**	652
**Cameroon**	43,232	**Mozambique**	89,943
**Central African Republic**	6,768	**Namibia**	22,754
**Chad**	11,267	**Niger**	881
**Comoros**	95[a]	**Nigeria**	188,660
**Congo**	7,191	**Rwanda**	8,349
**Côte d'Ivoire**	20,654	**Sao Tome and Principe**	16
**Democratic Republic of the Congo**	23,010	**Senegal**	960
**Equatorial Guinea**	708	**Sierra Leone**	2,972
**Eritrea**	342	**South Africa**	299,222
**Ethiopia**	19,533	**South Sudan**	16,672
**Gabon**	1,090	**Swaziland**	11,180
**Gambia**	1,473	**Togo**	3,752

**Ghana**	9,475	**Uganda**	92,403
**Guinea**	8,650	**United Republic of Tanzania**	50,357
**Guinea-Bissau**	2,197	**Zambia**	62,072
**Kenya**	67,449	**Zimbabwe**	68,222
**Lesotho**	22,178		

[a]: Estimate

[b]: from 2015 India estimates report

[c]: from 2013 estimates
