# Supplementary material for: The case for investing in the male condom
Source: PLoS One. 2017 May 16;12(5):e0177108. doi: 10.1371/journal.pone.0177108 (PMC5433691; doi:10.1371/journal.pone.0177108)
Supplement: S5 Table — (PDF) [file pone.0177108.s006.pdf]

S6 Table. Effectiveness of male condoms against STI incidence

STI	Incidence[a] reduction (cumulated over follow-up period)		Source & Comments
	Men	***Women***	
Gonorrhea	56%	56%	[5]
Chlamydia	38%	38%	[5]
Syphilis	29%	29%	[5]
HSV-2	29%	29%	Quantitative meta-analysis estimated the incidence reduction (among the small subset of yet uninfected women) as high as on average 61% [5, 16], but in light of qualitative evidence that condoms are less effective against HSV-2 (which spreads beyond the genital area) than for bacterial STIs, we conservatively put effectiveness as the lowest among the other STIs (syphilis).
Trichomoniasis	0%	0%	[5]: Condoms may protect women partially or not at all; not protection for men
HPV-2	0%	0%	[5]; no effect on incidence of infection, but couples randomized to condoms had higher rates of disease regression and HPV clearance.

[a] Incidence here is defined as episodes averted for gonorrhea, chlamydia and syphilis; and new infections averted (among the subset of HSV-uninfected people) for HSV-2.
