# Supplementary material for: The case for investing in the male condom
Source: PLoS One. 2017 May 16;12(5):e0177108. doi: 10.1371/journal.pone.0177108 (PMC5433691; doi:10.1371/journal.pone.0177108)
Supplement: S6 Table — (PDF) [file pone.0177108.s007.pdf]

S7 Table. Baseline STI incidence rates (per 1000 person-years 15-49 years) against which condom effectiveness was applied

Region	***Gonorrhea***		***Chlamydia***		***Syphilis***		***HSV-2***	
	Men	***Women***	***Men***	***Women***	***Men***	***Women***	***Men***	***Women***
AFRO	17	38	26	32	4.4	4.3	14.17	15.66
AMRO/PAHO	25	18	29	72	1.9	1.9	6.17	9.76
EMRO	17	13	33	31	1.5	1.5	1.17	3.80
EURO	12	8	20	19	1.0	1.0	2.24	4.07
SEARO	16	9	12	15	0.9	0.9	2.96	5.39
WPRO	46	33	64	56	1.0	1.0	4.03	5.49
Source	[19]		[19]		[19]		[20]	

Note: Estimates were for the year 2012, but assumed to be applicable in 2015 still, in line with assumptions about STI burden trends used in WHO's global STI strategy costing of September 2015. For HSV-2, incidence indicates new infections (not recurrent ulcer episodes due to HSV-2).
