# Supplementary material for: The case for investing in the male condom
Source: PLoS One. 2017 May 16;12(5):e0177108. doi: 10.1371/journal.pone.0177108 (PMC5433691; doi:10.1371/journal.pone.0177108)
Supplement: S7 Table — (PDF) [file pone.0177108.s008.pdf]

S8 Table. Calculation and assumptions on DALYs per STI episode

**Metric**	**Age group**	**Source**	**Chlamydia**	**Gonorrhoea**	**Syphilis**	**Genital herpes & HSV-2**
Prevalent episodes	15-49y	WHO 2012/2015 & [20] for HSV-2	126,719,000	28,565,000	17,695,000	417,000,000
Incident episodes	15-49y	WHO 2012/2015	130,107,147	83,514,235	5,589,625	21,300,000
DALYs	All ages	GBD 2013/2015	692,400	313,900	11,324,500	311,600
DALY per incident episode		Avenir's derivation	0.0053	0.0038	2.0	0.0146
Comments					DALYs reflect tertiary syphilis only	DALYs do not account for congenital herpes
