# Supplementary material for: The case for investing in the male condom
Source: PLoS One. 2017 May 16;12(5):e0177108. doi: 10.1371/journal.pone.0177108 (PMC5433691; doi:10.1371/journal.pone.0177108)
Supplement: S8 Table — (PDF) [file pone.0177108.s009.pdf]

S9 Table. Parameters used in the sensitivity analysis, and range of values

		Sensitivity Range	
**Parameter**	**Base**	**Low**	**High**
Unit cost of condom distribution	Base value	75% of base	125% of base
Coital frequency for family planning users	120	70	120
Coital frequency for HIV/STI risk groups			
SW	220	170	270
MSM	100	70	120
Transgenders	100	70	120
Prisoners	20	10	30
Multiple partners	100	70	120
Sero-discordant couples	70	70	100
Discount rate for costs	3%	0%	6%
Child DALYs averted as part of family planning impact	Not included	Included	
